# Appropriateness of Alerts and Physicians’ Responses With a Medication-Related Clinical Decision Support System: Retrospective Observational Study

**DOI:** 10.2196/40511

**Published:** 2022-10-04

**Authors:** Hyunjung Park, Minjung Kathy Chae, Woohyeon Jeong, Jaeyong Yu, Weon Jung, Hansol Chang, Won Chul Cha

**Affiliations:** 1 Department of Digital Health Samsung Advanced Institute of Health Sciences & Technology Sungkyunkwan University Seoul Republic of Korea; 2 Department of Emergency Medicine Samsung Medical Center Sungkyunkwan University School of Medicine Seoul Republic of Korea; 3 Digital Innovation Center Samsung Medical Center Seoul Republic of Korea

**Keywords:** clinical decision support system, computerized physician order entry, alert fatigue, health personnel, decision-making support, physician behavior, physician response, alert system

## Abstract

**Background:**

Alert fatigue is unavoidable when many irrelevant alerts are generated in response to a small number of useful alerts. It is necessary to increase the effectiveness of the clinical decision support system (CDSS) by understanding physicians’ responses.

**Objective:**

This study aimed to understand the CDSS and physicians’ behavior by evaluating the clinical appropriateness of alerts and the corresponding physicians’ responses in a medication-related passive alert system.

**Methods:**

Data on medication-related orders, alerts, and patients’ electronic medical records were analyzed. The analyzed data were generated between August 2019 and June 2020 while the patient was in the emergency department. We evaluated the appropriateness of alerts and physicians’ responses for a subset of 382 alert cases and classified them.

**Results:**

Of the 382 alert cases, only 7.3% (n=28) of the alerts were clinically appropriate. Regarding the appropriateness of the physicians’ responses about the alerts, 92.4% (n=353) were deemed appropriate. In the classification of alerts, only 3.4% (n=13) of alerts were successfully triggered, and 2.1% (n=8) were inappropriate in both alert clinical relevance and physician’s response. In this study, the override rate was 92.9% (n=355).

**Conclusions:**

We evaluated the appropriateness of alerts and physicians’ responses through a detailed medical record review of the medication-related passive alert system. An excessive number of unnecessary alerts are generated, because the algorithm operates as a rule base without reflecting the individual condition of the patient. It is important to maximize the value of the CDSS by comprehending physicians’ responses.

## Introduction

### Background

Computerized physician order entry (CPOE), linked to a clinical decision support system (CDSS), has become essential in the health care system. The main purpose of a CDSS is to improve patient safety and quality of care, and a medication-related CDSS is especially valuable [1,2]. In a medication-related CDSS, the alerting system provides dosing guidance or drug-drug, drug-allergy, and drug-age warnings that help clinicians prescribe correct orders. Early studies on CDSSs prompted substantial anticipation that medication-related CDSSs, such as alerting systems, may prevent adverse events and enhance patient safety [3,4].

Despite the increasing implementation of CDSS alerts, a substantial number of alerts are overridden [5-7]. The alert override rate is high, sometimes up to 96% [5]. Override is often invoked for reasons such as low alert specificity (ie, a lack of clinical relevance) and inadequate alert content [8,9]. Low alert acceptance was associated with repeated alerts that are inappropriate [6,10]. Excessive alerts that are not clinically relevant could lead to alert fatigue and contribute to alert overrides [11,12].

A common issue connected with the implementation of clinical decision support tools in electronic medical records (EMRs) is alert fatigue [13]. Alert fatigue is the issue in which users of a CDSS that generates an excessive amount of warning messages tend to overlook the majority of these alerts, including those that warn them of potentially clinically relevant errors [2]. A CDSS can fail to enhance patient safety due to alert fatigue. Alert fatigue arises when an excessive number of irrelevant alerts drives users to routinely override them [14]**.**

In the CDSS, 2 types of alerts are usually used. One type of alerts is active or “pop-up” warnings. These alerts require an action from the user for the clinical process to continue, such as clicking a button or stating the overriding reason. The other type of alerts is passive warnings, such as flagging potentially abnormal values. Passive alerts, unlike active alerts, do not interrupt the provider’s workflow; hence, these alerts do not require a response from the user to override the clinical process. Numerous studies have established the issue of alert fatigue with active alerts [10,12,15,16]. Passive alerts may also be a substantial cause of alert fatigue. The true burden of these alerts has rarely been assessed [17].

There is limited research evaluating the appropriateness of overrides with no override reasons in the passive alert system and the alert itself for clinical appropriateness for a patient’s specific condition. To understand the behavior of physicians, previous studies have only evaluated the appropriateness of overrides based on their reasoning [1,18]. In this study, we evaluated the appropriateness of alerts and physicians’ responses in a passive alert system through a patient EMR. We also categorized the alerts assessed by clinical relevance and physicians’ responses. This study may provide insights into the clinical use of medication alerts, whether physicians override them, and what reactions physicians offer when responding to them.

### Objective

This study aimed to evaluate the clinical appropriateness of alerts and the corresponding physicians’ responses in a medication-related passive alert system.

## Methods

### Study Design

This study was a retrospective observational study with stratified sampling according to medication. The analyzed alerts were generated from medication orders between August 2019 and June 2020 in the emergency department (ED). We obtained medication orders, alerts, and patient EMR data from a clinical data warehouse (CDW). In Korea, it is stipulated by law that only physicians can prescribe orders, except in a limited number of cases.

### Ethics Approval

This study was approved by the Institutional Review Board of the Samsung Medical Center (IRB 2021-09-115).

### Study Setting

This study was conducted in the ED of a tertiary academic medical center in Seoul, Korea. It serves 2 million outpatient visits annually and provides in-hospital service for 1975 beds. The ED has 69 beds and approximately 35 doctors. The annual number of patients visiting the ED ranges from 75,000 to 80,000. The workflow of the ED is uncontrolled and unpredictable [19]. Adverse events following an ED visit were reported less frequently but were more preventable than in other hospital settings [20]. Since the ED has various medication prescription patterns, diverse alerts can be analyzed by checking the patients in the ED.

### EMR System and Medication Order (Prescription) System

Our EMR system is a self-developed system implemented in 2016. Data Analytics and Research Window for Integrated Knowledge (DARWIN) is an extensive system that includes CPOE as well as nursing, pharmacy, billing, and research support and even patient portal and web services.

### CDSS Design: Passive Alert System

A passive alert system in the medication CDSS was applied to the DARWIN. Although passive alerts with in-line text do not interfere with physicians’ workflow, they may also result in decreased effectiveness of the CDSS alerts [21]. The alert appears before the order is confirmed. A response is not required to allow the prescription. The rule-based database for the CDSS was supplied by the KIMS POC knowledge base (KIMS Co) with weekly updates. The types of alerts were age, allergy, dose, drug-drug interaction (DDI), and renal.

### CDW Use

This study was performed using data extracted from the CDW at the study site. The CDW is an integrated storage for clinical data that are updated daily, such as deidentified patient demographic information, diagnosis, prescription, and laboratory results. In the past, researchers had to check the variables required for research individually and process the data accordingly. However, using the CDW, researchers can easily obtain the data automatically, sorted according to the various variables assumed by the researcher. CDW supports the automatic conversion of unstructured data, such as text to standardized data, to make it possible to conduct prospective cohort studies conveniently.

### Selection of Alerts

In all, 20 frequently overridden medication alerts were selected. We thought that alerts that are frequently overridden would be less clinically relevant; therefore, we prioritized alerts that are frequently overridden as evaluation targets. DDI types and alerts that are difficult to evaluate for clinical appropriateness were excluded as follows: when there was no specific dose setting information for reduction and when the range of dose adjustment according to the indication and severity was wide. Overridden cases and nonoverridden cases were randomly extracted from 20 frequently overridden medication alerts. The number of cases for each medication alert are shown below.

### Definition of Alert Overrides and Appropriateness

Alert overrides occur when physicians do not change orders as suggested by the alert. Our previous study defined an alert override as no change in order when an alert occurred on the log data [22]. In this study, however, alert override means no change in order when an alert occurred or a re-order of the same prescription later. In nonoverridden cases, many physicians prescribed the nonoverridden order again, and we considered this case to be an override. If the identical prescription that generated the alert was given to the same patient within 48 hours, it was deemed an override. Alert clinical relevance means that the alert is suitable for each patient’s condition and that the alert actually helped the physician order the prescription. The physicians’ response appropriateness indicates whether the physicians’ override or nonoverride was appropriate considering the patient’s clinical condition.

### Detailed Medical Record Review

Through advanced medical record reviews of alert overridden cases and literature research, a group of 3 clinicians (a physician, a pharmacist, and a nurse) determined the criteria for the appropriateness of each alert. In a detailed medical record review, information such as the patient’s age, gender, weight, laboratory results (potassium, sodium, serum creatinine, or glomerular filtration rate, etc), and computed tomography status was confirmed through the patient’s EMR. Each group member independently reviewed random samples of the 382 alert cases for the evaluation of the appropriateness of alert clinical relevance and physicians’ responses. When panel members disagreed, consensus was reached via group discussion.

### Classification of Alerts

**Figure 1:**
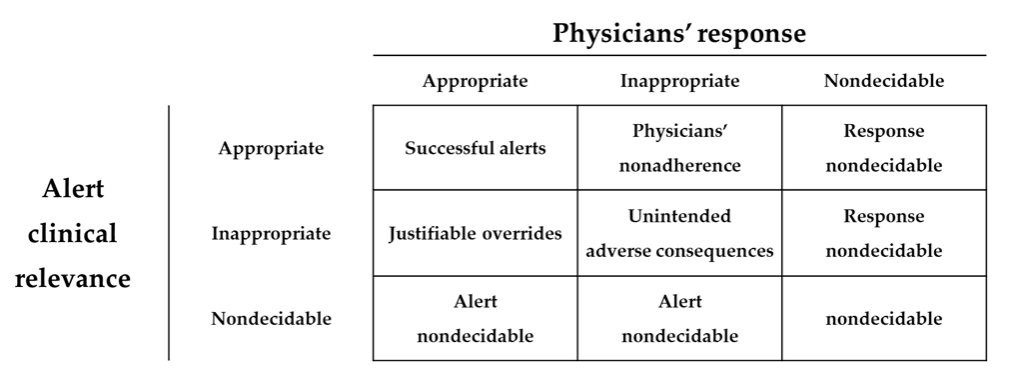
Classification table for alerts. The alert classification table included the nondecidable category—since the passive alert system does not include an override reason, some cases might be difficult to evaluated.

The alerts were classified based on the results of the appropriateness evaluation. We referred to the evaluation framework developed by McCoy et al [23]. Since the passive alert system does not collect the overriding reason, it may be difficult to judge the appropriateness. Therefore, we included a nondecidable category in the alert classification table (Figure 1).

### Korean Triage and Acuity Scale (KTAS)

The KTAS is an evaluation tool used to categorize the severity and urgency of ED patients. It is a 5-level triage scale based on the severity of the patient’s chief complaint and symptoms. The KTAS was established in 2012 in Korea in an effort to enhance patient safety and minimize ED congestion at the hospital level. Patients who enter the ED are evaluated by KTAS using the following procedure: impression evaluation, infection confirmation, primary symptom selection, and primary/secondary considerations [24,25].

### Data Analysis

Commonly overridden medications were subgrouped according to alert type, and alert patterns were examined. Samples for the medical record review were extracted using stratified random sampling. In our samples, we analyzed the appropriateness of alerts, physicians’ responses, and patient demographics. Interrater reliability for the evaluation of alert and physicians’ response appropriateness was calculated by using a κ index. The results are presented as counts and percentages. The rate of false positive alerts, physicians’ response inappropriateness, and override were expressed as percentages of total alerts. All statistical tests were performed using R statistical software (version 4.0.3; R Foundation for Statistical Computing).

## Results

**Figure 2:**
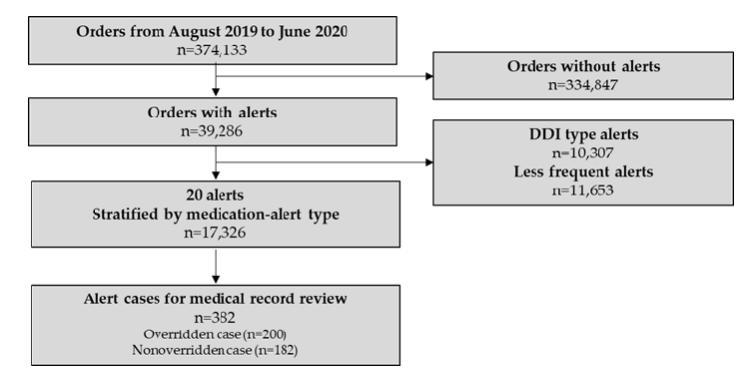
Study flow chart. DDI: drug-drug interaction.

Figure 2 shows the detailed selection process for medication alert data. A total of 39,286 (10.5% alert rate) CDSS alerts occurred for 374,133 medication orders between August 2019 and June 2020. We selected 20 frequently overridden medication alerts stratified by the medication alert type (Table 1). The number of alert cases analyzed for medical record reviews was 382 (200 overridden and 182 nonoverridden cases).

The medical record review included 356 patients. Table 2 shows the demographic information of the patients in the medical record review cases. Overall, the patients’ basic characteristics showed that the majority were men (204/356, 57.3%), aged more than 60 years (205/356, 57.6%), and had KTAS scores of 3 (197/356, 55.3%).

A total of 728 medications triggered an alarm; however, we chose 20 frequently overridden medication alerts, because we thought that alerts that are frequently overridden would be less clinically relevant. Table 1 shows the 20 analyzed medications. In the overridden case, all medication alerts included 10 cases; however, in the nonoverridden case, methylprednisolone (n=6), epinephrine (n=9), cefditoren (n=2), cefazolin (n=6), and ampicillin/sulbactam (n=9) had fewer than 10 cases.

Table 3 shows the results of the appropriateness evaluation for alert clinical relevance and physicians’ responses. Interestingly, of the 382 alert cases, the only 7.3% (n=28) were clinically relevant alerts. In the physicians’ response assessment, 92.4% (n=353) were appropriate and 1.6% (n=6) were nondecidable. The interrater reliability for alert clinical relevance appropriateness and physicians’ response appropriateness were moderate (κ=0.47) and fair (κ=0.28), respectively. In our study, there was no difference in the appropriateness of clinical relevance between overridden and nonoverridden alerts. When an overridden alert and a nonoverridden alert were classified using a data log rather than a medical record review, the alert appropriateness was 7% (14/200) for overridden alerts and 7.7% (14/182) for nonoverridden alerts, which did not show clinical relevance. Contrary to the expectation that there were more inappropriate alerts in nonoverridden alerts, there was no difference in alert appropriateness between the 2 types of alerts (Multimedia Appendix 1).

In the classification of the 382 alerts, only 3.4% (n=13) were successfully triggered, and 2.1% (n=8) were inappropriate for both the alert and physicians’ response (Table 4). Only 3.9% (n=15) of alerts represented physicians’ nonadherence, where the alert was appropriate but the corresponding physicians’ response was inappropriate. The override rate was 92.9% (n=355): *(Physicians’ nonadherence [n=15] + justifiable overrides [n=340]) / total alerts [n=382]* (Table 4). There were 6 (1.6%) cases in which the physicians’ response could not be determined.

**Table 1:** The 20 analyzed medication alerts.

Order (medication type)	Alert type	Alert counts, n	Overridden alerts for medical record reviews (N=200), n	Nonoverridden alerts for medical record reviews (N=182), n
Sodium bicarbonate, 8.4%, 20 mL (other)	Dose	2125	10	10
Esomeprazole, 40 mg (proton pump inhibitor)	Dose	1885	10	10
Ceftriaxone sodium, 2 g (antibiotic)	Renal	1379	10	10
Kalimate powder, 5 g (other)	Dose	1494	10	10
Tazoferan, 2.25 g (antibiotic)	Renal	1108	10	10
Calcium gluconate, 2 g/20 mL (calcium)	Dose	1230	10	10
Acetaminophen, 1 g/100 mL (analgesic)	Dose	1527	10	10
Pantoprazole, 40 mg (proton pump inhibitor)	Dose	1059	10	10
Lactulose syrup (other)	Dose	701	10	10
Propacetamol, 1 g (analgesic)	Age	1205	10	10
Methylprednisolone, 4 mg (steroid)	Dose	378	10	6
Ibuprofen, 20 mg/mL (NSAIDs^a^)	Dose	611	10	10
Levofloxacin, 750 mg (antibiotic)	Renal	421	10	10
Terlipressin acetate, 1 mg (vasoconstrictor)	Dose	386	10	10
Epinephrine, 1 mg (other)	Dose	340	10	9
Amiodarone, 150 mg (antiarrhythmic)	Dose	329	10	10
Meropenem, 500 mg (antibiotic)	Renal	301	10	10
Ampicillin/sulbactam, 1.5 g (antibiotic)	Dose	271	10	9
Cefazolin, 1 g (antibiotic)	Dose	275	10	6
Cefditoren pivoxil, 100 mg (antibiotic)	Dose	301	10	2

^a^NSAID: nonsteroidal anti-inflammatory drug.

**Table 2:** Patient demographic.

Demographic				Patient (N=356), n (%)
**Sex, n (%)**				
	Female		152 (42.7)	
	Male		204 (57.3)	
**Age (years), n (%)**				
	0 to 20		58 (16.3)	
	20 to <40		18 (5.1)	
	40 to <60		75 (21.1)	
	≥60		205 (57.6)	
**KTAS^a^ score, n (%)**				
	1 (most critical)		13 (3.7)	
	2		51 (14.3)	
	3		197 (55.3)	
	4		94 (26.4)	
	5 (least critical)		1 (0.3)	
**Injury, n (%)**				
	Noninjury		68 (19.1)	
	Injury		288 (80.9)	
**Disposition, n (%)**				
	Discharge		121 (34)	
	**Admission**		193 (54.2)	
		General ward (n=193)	165 (85.5)	
		Intensive care unit (n=193)	28 (14.5)	
	Transfer		22 (6.2)	
	Death		20 (5.6)	

^a^KTAS: Korean Triage Acuity Scale.

**Table 3:** Appropriateness of alert clinical relevance and physicians’ response.

Appropriateness evaluation	Case (N=382), n (%)		
	Appropriate	Inappropriate	Nondecidable
Alert clinical relevance	28 (7.3)	354 (92.7)	0 (0)
Physicians’ response	353 (92.4)	23 (6)	6 (1.6)

**Table 4:** Evaluation of alerts.

Alert clinical relevance	Physicians’ response (N=382), n (%)		
	Appropriate	Inappropriate	Nondecidable
Appropriate	13 (3.4)^a^	15 (3.9)^b, c^	0 (0)
Inappropriate	340 (89)^c^	8 (2.1)^d^	6 (1.6)
Nondecidable	0 (0)	0 (0)	0 (0)

^a^Successful alerts.

^b^Physician’s nonadherence.

^c^The override rate (355/382, 92.9%) was determined by the sum of these 2 values divided by the total number of alerts.

^d^Unintended adverse consequences.

## Discussion

### Principal Findings

In this study, we evaluated the appropriateness of the alerts and physicians’ responses to the medication-related passive alert system through a detailed medical record review. We found that only 7.3% of alerts were clinically appropriate, and 6% of alerts resulted in inappropriate responses from physicians. Alert fatigue is inevitable when a large number of irrelevant alerts are generated for a small number of appropriate alerts. There were a few successful alerts where the alert was appropriate and the physician accepted the alert. Physicians’ nonadherence of alerts could be a result of the ambiguous contents of alerts that did not provide helpful information [26]. Additionally, a high number of inappropriate alerts could be a reason for physicians’ nonadherence [27]. Physicians were less likely to accept alerts as the number of alerts increased, especially for repeated alerts [6]. When considering the cases where the response of the physician was inappropriate, the alerts where the alert was appropriate were almost twice as common as the alerts where the alert was inappropriate. This finding can be explained by habitual override due to numerous inappropriate alerts [28]. A small number of alerts were classified as resulting in unintended adverse consequences. In a few cases, the physicians’ response appropriateness could not be determined, because the passive alert system did not collect the override reasons. There were no cases where the appropriateness of the alert could not be determined.

Many studies have identified the appropriateness of override according to the appropriateness of the alert [1,5,15,29,30], but only a few studies have evaluated the response of physicians [31-33]. Duke et al [31] conducted a randomized controlled trial on DDI alert targets to identify medical staff’s adherence according to context-enhanced alerting. Strom et al [32] analyzed the unintended effects of a nearly hard-stop CPOE prescribing alert. Understanding the physicians’ response to the CDSS is of importance; however, due to the difficulty in analyzing the response, many researchers simply evaluate the appropriateness of the override. Therefore, it is necessary to increase the utility of the CDSS by understanding physicians’ responses.

In our previous study, we reported an override rate of 61.9% [22]. However, in this study, we found that the override rate was 92.9%. There are several reasons for this difference. First, in this study, through medical record reviews, it was confirmed that some cases that were previously evaluated as nonoverridden by log data were clinically overridden. The difference between the override rate when simply using log data and the override rate through a medical record review is large, even within the same system. In this study, the patients’ overall prescriptions were analyzed through a detailed patient medical record review, and the definition of “override” was expanded. In the previous study, the classification of overridden and nonoverridden alerts was based only on log data [22]. In this study, however, more override was detected by the medical record review than in the previous study. It was confirmed that a substantial number of cases classified as nonoverridden by log data were actually overridden. We found that many physicians prescribed the same prescription that was considered deleted because of an alert. The prescription was considered an override if it was reissued to the same patient within 48 hours of the alert being issued. Therefore, the override rate might be higher in studies that did not identify the nonoverridden alerts [15,29,34,35]. To calculate the override rate properly, it is necessary to establish a mechanism for systematically determining overrides. A standardized definition of override is needed for a detailed analysis and comparison of CDSSs. Furthermore, in this study, we chose the target alerts as alerts that are frequently overridden, so it could be a reason for the high override rate. Additionally, the change of the knowledge base of the CDSS from Medi-Span (Wolters Kluwer Health) to KIMS POC (KIMS Co) may have affected the override rate.

Further research should investigate techniques for improving alert accuracy by using machine learning (ML) and artificial intelligence (AI), analyze the passive CDSS that has not been extensively studied, and explore the causal relationship between the number of alerts and the physicians’ responses. Multiple alerts with low clinical relevance reduce physicians’ reliance of alerts. Additionally, many unnecessary alerts can lead to alert fatigue and increase the probability of ignoring truly important alerts [2]. It is necessary to improve the clinical relevance of the alert to increase the physician’s alert reliance and optimize the alert. ML and AI could be potential solutions. By introducing ML, the rule-based alert system can be improved, and by introducing AI, alerts can be generated according to the individual condition of the patient [36,37]. Despite the promise of technological approaches to drug safety, the risk of mistake will persist if these systems are not carefully applied and heavy attention is not made to building safer systems of care [2]. These considerations are required to reduce needless alerts, improve their clinical relevance, and increase physicians’ alert reliance by assessing CDSS consistently.

### Limitations

Our study had several limitations. First, it was performed at a single center with ED practices. Second, the evaluation of physicians’ response appropriateness may be subjective, because passive alert systems do not collect the override reasons. In addition, we did not confirm the clinical consequences of alerts for unintended adverse consequences. Only the clinical consequences related to the prescription stage were checked, and the dispensing/administration stage was not analyzed.

### Conclusions

We evaluated the appropriateness of the alerts and physicians’ responses through a detailed medical record review of the medication-related passive alert system. Only by gaining better knowledge of the physicians’ overall behavior is it possible to improve the effectiveness of the CDSS. In our study, most alerts did not reflect the clinical situation of each patient; however, the physicians’ responses were mostly appropriate. Alert fatigue is unavoidable when a large number of irrelevant alerts are generated in response to a small number of useful alerts. It is necessary to decrease unnecessary alerts, improve their clinical relevance, increase alert reliability, and optimize alerts.

## Appendix

Multimedia Appendix 1 Comparison of alert appropriateness according to overridden and nonoverridden alerts. There was no difference in the appropriateness of clinical relevance between overridden alerts (7% appropriate) and nonoverridden alerts (7.7% appropriate).

## Abbreviations

AI: artificial intelligence
CDSS: clinical decision support system
CDW: clinical data warehouse
CPOE: computerized physician order entry
DARWIN: Data Analytics and Research Window for Integrated Knowledge
DDI: drug-drug interaction
ED: emergency department
EMR: electronic medical record
KTAS: Korean Triage and Acuity Scale
ML: machine learning
