# Supplementary material for: Appropriateness of Alerts and Physicians’ Responses With a Medication-Related Clinical Decision Support System: Retrospective Observational Study
Source: JMIR Med Inform. 2022 Oct 4;10(10):e40511. doi: 10.2196/40511 (PMC9579928; doi:10.2196/40511)
Supplement: Multimedia Appendix 1 [file medinform_v10i10e40511_app1.docx]

**Multimedia Appendix 1.** Comparison of alert appropriateness according to overridden alerts and nonoverridden alerts.

|  | Overridden alert (N=200), n (%) | Nonoverridden alert (N=182), n (%) |
| --- | --- | --- |
| Appropriate alert | 14 (7) | 14 (7.7) |
| Inappropriate alert | 186 (93) | 168 (92.3) |
